# THE ROLE OF CARDIOVASCULAR MORTALITY IN LIFE EXPECTANCY GAPS AFTER AGE 50 BETWEEN US AND OTHER HIGH-INCOME COUNTRIES

**DOI:** 10.1093/geroni/igae098.2150

**Published:** 2024-12-31

**Authors:** Octavio Bramajo, Neil Mehta

**Affiliations:** The University of Texas Medical Branch, Galveston, Texas, United States; The University of Texas Medical Branch, Galveston, Texas, United States

## Abstract

Compared to other high-income countries, the United States underperforms in life expectancy. One key driver of this differential is cardiovascular disease (CVD) mortality, a cause of which the US has had worse trends since around 2008. The differential in CVD mortality has been previously described, however, its contribution to the widening of the US life expectancy lag relative to other high-income countries is unknown. We measured the contribution of CVD mortality to the post-2010 life expectancy divergence between the US and other ten high-income countries. Data and

**methods:**

Using life table methods and WHO cause-specific mortality data, we computed life expectancy at age 50 (LE50) by sex and by two counterfactual scenarios to establish the contribution of CVD mortality in the growth LE50 gap between 2008 and 2019: first in a cause-deleted approach, removing CVD deaths, and by replacing the age-specific CVD mortality rates of the high-income countries in 2019 with the corresponding US CVD mortality improvement rate. The US had a lower LE50 compared to the average of other high-income countries in 2008), and the LE50 gap with those countries increased in 2019 by 0.4 years for females and 0.8 years for males. Either removing or replacing CVD mortality resulted in narrowing the overall LE50 gap between the US and the high-income countries in 2008 and 2019, and in most cases doing so was able to close it entirely by themselves, especially for females, with CVD explaining entirely the growth of the gap

